# Flour Treatments Affect Gluten Protein Extractability, Secondary Structure, and Antibody Reactivity †

**DOI:** 10.3390/foods13193145

**Published:** 2024-10-02

**Authors:** Bruna Mattioni, Michael Tilley, Patricia Matos Scheuer, Niraldo Paulino, Umut Yucel, Donghai Wang, Alicia de Francisco

**Affiliations:** 1 Department of Biological and Agricultural Engineering, Kansas State University, Manhattan, KS 66506, USA; dwang@ksu.edu; 2 USDA, United State Department of Agriculture, Agricultural Research Service Center for Grain and Animal Health Research, 1515 College Avenue, Manhattan, KS 66502, USA; michael.tilley@usda.gov; 3 Federal Institute of Santa Catarina, IF-SC, Rua 14 de Julho, 150, Coqueiros, Florianopolis 88075-010, SC, Brazil; patriciamatosscheuer@hotmail.com; 4 MEDICAL LEX Information Management and Educational Courses S.A. Vitor Lima 260 Sala 908, Ed. Madson Center Trindade, Florianopolis 88040-400, SC, Brazil; niraldop@yahoo.com.br; 5 Department of Food, Nutrition, Dietetics and Health, Kansas State University, Manhattan, KS 66506, USA; yucel@ksu.edu; 6 Laboratory of Cereals, Food Science and Technology Department, Federal University of Santa Catarina, Av. Admar Gonzaga, 1346, Itacorubi, Florianopolis 88034-001, SC, Brazil; aliciadf@gmail.com

**Keywords:** wheat, glutenin, gliadin, celiac disease

## Abstract

Commercial Brazilian wheat flour was subjected to extrusion, oven, and microwave treatments. The solubility, monomeric and polymeric proteins, and the glutenin and gliadin profiles of the gluten were analyzed. In addition, in vitro digestibility and response against potential celiac disease immune-stimulatory epitopes were investigated. All treatments resulted in low solubility of the polymeric and monomeric proteins. The amounts of insoluble proteins increased from 5.6% in control flour to approximately 10% for all (treatments), whereas soluble proteins decreased from 6.5% to less than 0.5% post treatment. In addition, the treatments affected glutenin and gliadin profiles. The amount of α/β-gliadin extracted decreased after all treatments, while that of γ-gliadin was unaffected. Finally, the potential celiac disease immune stimulatory epitopes decreased in oven and microwave treatment using the G12 ELISA, but no change was observed using the R5 antibody. However, the alteration of the gluten structure and complexity was not sufficient to render a product safe for consumption for individuals with celiac disease; the number of potential celiac disease immune-stimulatory epitopes remained high.

## 1. Introduction

Wheat is one of the most important staple crops in temperate areas worldwide and is an important source of nutrients for millions of people [1]. A large variety of baked products can be made from wheat flour because of its ability to form viscoelastic dough [2]. This is primarily attributed to the gluten proteins [3]. These are among the most complex proteins in nature owing to their various components and sizes, ranging from dimers to polymers, with molecular weights exceeding one million kDa. Their variability is caused by genotype variants, growth conditions, and technological processes [4]. Gluten proteins play a key role in determining the unique rheological dough properties and baking quality of wheat [3,5,6].

Gliadins and glutenins are the two main classes of gluten protein that determine the technological characteristics of wheat flour [3]. They are classified according to their solubility, molecular weight, and electrophoretic mobility. Gliadins are monomeric proteins that are soluble in alcohol. They are categorized into α, β, γ, ω-gliadin, and sulfur-rich or -poor (S-rich or S-poor) gliadins, with molecular weights ranging from 30 to 74 kDa [4]. Glutenins are polymeric proteins and are classified according to their molecular weight into high (HMW-GS) (80–160 kDa) and low molecular weight (LMW-GS) (30–51 kDa), and genotype (x or y). There are 7 to 16 different LMW-GS in each genotype and these can be classified according to their N-terminal amino acids such as i-, s-, and m-LMW-GS (isoleucine, serine, and methionine, respectively) [4]. The quantity of HMW-GS is strongly correlated with dough properties and bread quality [7,8].

The unique structure of the gluten network is due mainly to covalent (disulfide) and noncovalent (hydrogen, ionic, and hydrophobic) bonds, owing to their amino acid composition which has a high amount of glutamine and proline as well as low levels of charged amino acids [2,9].

Treating wheat with processes involving temperature and pressure can change its protein structure. Heat processing can affect technological properties and reduce allergenicity to wheat flours and breads to varying extents [10]. Protein degradation occurs with increasing temperature and mainly involves cysteine and lysine amino acids [9]. During dough preparation and baking, competitive redox reactions occur in the glutenin polymer network: (1) the oxidation of free SH groups which supports polymerization; (2) chain ‘terminators’ that stop polymerization, and (3) SH/SS interchange reactions between glutenins and thiol compounds such as glutathione that depolymerizes polymers [2,11]. Another important production process is extrusion, in which high temperature, pressure, screw speed, shear, die geometry, and moisture content result in various low-density products, such as meat analogs, breakfast cereals, snacks, starches, and baby foods [12].

Immune-mediated diseases triggered by gluten consumption include celiac disease (CD), gluten ataxia, and dermatitis herpetiformis [13]. The primary trigger of the immune response in celiac disease (CD) is the specific gluten protein epitopes that are resistant to digestion. The most common symptoms of CD include malnutrition, diarrhea, growth retardation, anemia, and fatigue [14] resulting from inflammatory injury to the small intestine mucosa after gluten consumption [15]. Different gliadin types (α/β-type, ω-type, and γ-type gliadins) as well as glutenins [16,17] have been shown to have important and variable roles in the disease’s pathogenesis and inflammatory response [15,18]. Some researchers have theorized that heat treatment can affect the toxicity and chemical characteristics of gluten [19,20,21,22]. Therefore, this study aimed to provide a better understanding of the possible processing-induced changes in gluten proteins. We investigated how processing (extrusion, oven, and microwave) affects gluten protein network solubility, secondary protein structure, and the microstructure of the flour, and whether any treatment tested affected protein digestibility or celiac disease epitopes.

## 2. Materials and Methods

### 2.1. Sample Processing

All chemicals and reagents were of analytical grade, and all sample treatments and analyses were performed in triplicate. Brazilian commercial fortified white wheat flour (*Triticum aestivum*) was obtained from Cooperativa Agrária Agroindustrial (Guarapuava, PR, Brazil) and was analyzed before and after the following treatments:

Extrusion: Pilot-scale extrusion was performed under optimal operating conditions using a single-screw MX40 pilot extruder (Inbramaq, Ribeirão Preto, SP, Brazil). The extrusion conditions were modified based upon previous experience as follows: the temperature of the barrel head was 120 °C, there was a water addition of 30% (*w*/*v*) in relation to flour, and the screw speed was 220 rpm. The flow rate was approximately 20% of the nominal capacity and amounted to 50 kg/h. The L/D ratio was 2.3:1, the screw diameter was 92.5 mm, and the processing barrel length was 210 mm. The diameter of the ten circular nozzles was 3 mm. The dough feed rates to the screw and the barrel were 40 and 50%, respectively.

Dry heat oven: Dough was made by mixing 200 g of flour with a sufficient amount of water, as previously described [23]. The dough was hand kneaded until it passed the windowpane test, and approximately 40 g of dough was rolled to uniform thickness (3.0 mm) and placed in an oven at 250 °C for 5 min [24].

Microwave: Wheat flour was suspended in water 90% (*w*/*v*) and exposed to microwave radiation in a laboratory microwave for 5 min at 500 W [19].

After all treatments, the samples were lyophilized, ground with IKA (A 11 basic Analytical mill, IKA Works, Inc., Wilmington, NC, USA) and sieve to 0.5 mm sieve, then stored at −5 °C before conducting further analysis.

### 2.2. Scanning Electron Microscopy

The individual samples were mounted on stubs and secured using carbon tape, coated with a 350 Å gold layer, and examined in a JEOL JSM-6390LV scanning electron microscope (JEOL USA, Peabody, MA, USA). The working distance was set at 15 mm with a voltage of 10 kV.

### 2.3. Determination of Total Protein (%TP)—LECO

All samples were analyzed via nitrogen combustion using a Leco FP-428 nitrogen determinator (Leco, St. Joseph, MI, USA) according to the AACC method 46-30.01 [25]. A factor of N = 5.7 was used for protein determination.

### 2.4. Determination of Percentage of Insoluble Polymeric Protein (%IPP) and Monomeric and Soluble Polymeric Protein (%SPP)

Proteins were extracted according to the method described [26]. The extracted proteins were lyophilized, and the protein content was determined as described above. *SPP* (%) was determined by Equation (1):

(1) %SPP=%TP−%IPP

### 2.5. Determination of Monomeric and Polymeric Distribution—Size Exclusion HPLC

To determine the monomeric and polymeric distributions of wheat proteins, size exclusion high-performance liquid chromatography (SEC-HPLC) was carried out, as previously described [27].

Total polymeric protein (TPP), extractable polymeric protein (EPP), and unextractable polymeric protein (UPP) were extracted as described [27,28].

After extraction, analyses were performed using an Agilent 1100 HPLC instrument (Agilent, Palo Alto, CA, USA). The protein extract (20 µL) was injected into a BioSep-SEC s4000 analytical column (300 mm length × 7.8 mm ID, 5 um particle size, 500 Å pore size) (Phenomenex, Torrance, CA, USA) and run for 30 min on an isocratic gradient of 50% water containing 0.1% trifluoroacetic acid (TFA) and 50% acetonitrile containing 0.1% of TFA at a constant flow rate of 0.5 mL/min with a column temperature of 30 ºC. The post run lasted 10 min. Absorbance was measured at 210 nm using a variable wavelength detector. The relative molecular weight distributions of the polymeric proteins were obtained based on the method described [27].

### 2.6. Gliadin and Glutenin Profile—Reverse Phase HPLC (RP-HPLC)

Gliadin and glutenin were extracted as described by [29]. After extraction, the glutenins and gliadins were analyzed with RP-HPLC using an Agilent Technologies 1260 Infinity HPLC system (Agilent, Palo Alto, CA, USA). Extracts (20 µL injection) were analyzed using a Jupiter C18 analytical column with a 5 µm particle size and a 300 Å pore size (250 mm length × 4.6 mm ID) (Phenomenex, Torrance, CA, USA), and the eluent absorbance was measured using a UV detector at 210 nm.

For gliadins, proteins were eluted using the following solvents: (A) water containing 0.1% TFA, and (B) acetonitrile containing 0.05% TFA in a 25% to 50% linear gradient of B over 80 min at a constant flow rate of 1 mL/min. The column temperature was set to 70 °C with a 10 min post run.

For glutenins, proteins were eluted using solvents (A) and (B) in a 23–60% linear gradient of B over 40 min at a constant flow rate of 1 mL/min. The column temperature was set to 70 °C with a 10 min post run.

### 2.7. Fourier Transform Infrared (FTIR) Spectroscopy

The FTIR analysis was conducted using a Perkin Elmer FTIR with Attenuated Total Reflection (ATR) equipped with a single-bounce diamond crystal and a deuterated triglycine sulfate (DTGS) detector. Spectra were collected at room temperature, with a 400–4000 cm^−1^ range, a resolution of 4 cm^−1^, data spacing of 0.482 cm^−1^, and 64 scans. Each spectrum was corrected for a linear baseline over five points (ca. 4000, 3990, 2500, 1880, and 700 cm^−1^). The secondary protein structures were determined and quantified by deconvoluting the amide I band peak observed between 1600 and 1700 cm^−1^ using GRAMS/AI 9.2 software (Thermo Fisher Scientific, Waltham, WA, USA) following a second-order derivative approach, as described [30]. Briefly, the second-order derivative of the complex electron paramagnetic resonance (EPR) spectra was taken and enhanced using a Savitzky–Golay function, which was followed by a non-linear least squares peak fitting process, assuming a mixed Lorentzian and Gaussian wave distribution using the Voigt function. A double-subtraction protocol was applied to account for the water contribution. The first subtraction was performed automatically by the instrument to account for any residual water vapor in the air, and the second was performed using a water reference spectrum. The areas under each peak were used for quantification.

### 2.8. Standard In Vitro Protein Digestibility

The protein digestibility of all samples was determined using protocols previously described [31,32]. Undigested proteins were determined by nitrogen combustion (n × 5.7) using LECO. The digestibility was calculated using the following Equation (2):

(2) $$\%digestibility=\frac{P_{total}-P_{undigested}}{P_{total}\times 100}$$

where

*P_total_* = Total protein;

*P_undigest_* = Undigested protein.

### 2.9. Immunoreactivity Using ELISA R5 and G12

The processing effects on immunoreactivity were analyzed via ELISA, using R5 and G12 antibodies after gluten extraction. The flours were extracted using the Méndez Cocktail [33], followed by the addition of 80% ethanol to a final concentration of 60% ethanol.

ELISA R5: The extracted samples were analyzed using the R5 Method, as previously described [34]. Briefly, a Ridascreen Gliadin R5 sandwich ELISA kit (#7001 R-Biopharm Ag, Darmstadt, Germany) was used according to the manufacturer’s instructions.

ELISA G12: The extracted samples were analyzed using a G12 antibody-based sandwich ELISA test kit (AgraQuant^®^ Gluten G12 ELISA) from Romer Labs (Romer Labs, Union, MO, USA), according to the manufacturer’s instructions. Values are expressed as g of gluten/100 g flour.

### 2.10. Statistical Analysis

The results were expressed as mean ± standard deviation (SD). Bartlett’s test was used to verify the homogeneity of the variances. Differences in protein levels among the different treatment groups were determined using one-way analysis of variance (ONE-WAY ANOVA). Multiple comparisons were performed using Tukey’s post hoc test, and the criterion for significance was set at *p* < 0.05.

## 3. Results and Discussion

### 3.1. Scanning Electron Microscopy

**Figure 1:**
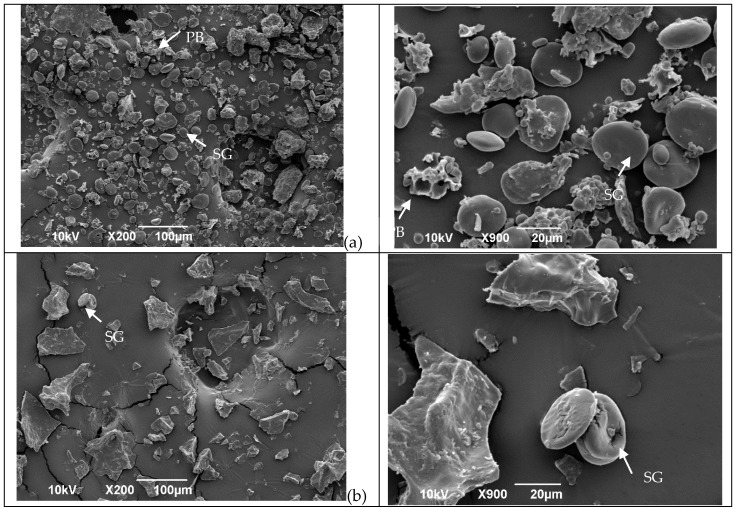

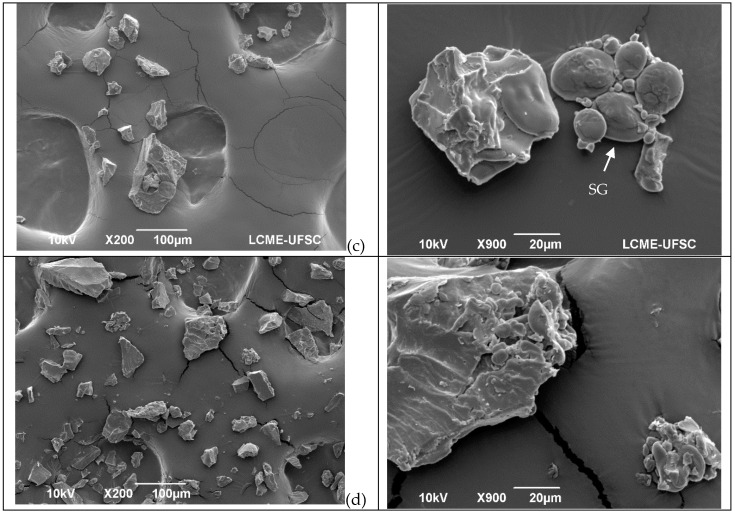
Microstructures of wheat flour before and after different treatments in two different magnitudes. (**a**) = Untreated flour; (**b**) = microwave; (**c**) = oven, and (**d**) = extrusion. Arrows in the micrographs indicate starch granules (SG) and protein bodies (PB) in the sample.

The SEM images in Figure 1 reveal the influence of the treatments (extrusion, oven, and microwave) on flour structure. In the control flour (Figure 1a), both type A and B starch granules appear with a smooth clean surface and are free from the protein matrix. The same results were observed by Scheuer, et al. [35]. However, after treatment (Figure 1b–d), the microstructure changed.

After flour extrusion, the starch granules were not easily detected because of starch gelatinization, resulting in a general homogenous and porous structure (Figure 1d). After microwave irradiation, the microstructure was compact, and the starch appeared to be gelatinized (Figure 1b). After kneading and oven treatment, the microstructure of the protein network structures was observed (Figure 1c) as a result of the progressive development of the viscoelastic properties of the dough [36,37], which occurs due to changes in the gluten protein polymer structure as both covalent and noncovalent bonds are reorganized, resulting in a complex continuous network that entraps starch and gas molecules [4].

### 3.2. Total Protein, Soluble and Insoluble Polymeric Protein

The treatments affected the solubility of the gluten proteins, as determined by the amount of polymeric and monomeric proteins. In all treatments the solubility of proteins decreased; the amount of IPP (insoluble polymeric protein) was higher in the treatment groups than that in the control flour (Table 1), with a concomitant decrease in SPP (soluble polymeric protein). These treatments involved mechanical work and/or high temperatures. An increase in temperature results in an alteration in protein conformation due to an increase in chemical interactions, including in covalent and noncovalent bonds that stabilize gluten structures, resulting in a decrease in solubility [38].

Treatment conditions had no effect on the total protein content (TP) (*p* < 0.05) (Table 1). The IPP values agreed with those reported in previous studies on wheat flour [37]. IPP (insoluble polymeric protein) is a protein quality indicator that correlates better than protein content with bread loaf volume, bake mix time, and mixing tolerance [26]. In terms of dough quality, a higher IPP content increases the retention of CO_2_, and bread dough becomes hard and less elastic. However, low IPP content is related to low elasticity and the weakening of the gluten network, and high SPP (soluble polymeric protein) content is related to the low extensibility strength of the dough [37].

As reported by Silvas-García et al. [37], changes in the IPP and SPP content indicate modifications in the gluten polymer chains. Therefore, heat treatment resulted in an increase in the molecular size of gluten polymers.

The hydrophobic interactions that occur during heating promote the formation of aggregates [39]. These bonds are different from other bonds because their energy increases with increasing temperature, which provides additional stability during baking [2]. The most important covalent bonds are disulfide, tyrosine, and hydrophobic bonds. Disulfide bonds play a significant role in determining the structure and properties of gluten proteins. Monomeric α/β- γ- and ω-gliadins have three and four intrachain disulfide bonds, respectively, whereas polymeric LMW- and HMW-GS have both intra- and interchain bonds [2].

As the temperature increased, the hydrophobic interactions increased, owing to the disruption of the ionic and hydrogen bonds in the gluten protein. These hydrogen bonds primarily contribute to holding the gluten dough together. Temperatures above 60 °C denature the gluten proteins, causing them to unfold and resulting in free SH groups that are susceptible to oxidation and intra- or intermolecular disulfide bond formation [39].

### 3.3. Total, Extractable and Unextractable Polymeric Proteins by Size Exclusion HLPC

SEC-HPLC is useful for obtaining information on the solubility of protein fractions induced using heat treatment and protein aggregation, and help to better understand the gluten network arrangement [40].

Our results showed a significant increase and decrease in the extractability of monomeric and polymeric proteins, respectively. Overmixing dough decreases the HMW-GS and gliadin extractability [41]. Ionic, S-S, and hydrogen bonds are affected by heat treatment, leading to the unfolding of wheat gluten [39]. These changes affect the secondary structure of gluten and influence the dough’s rheological properties [12].

**Figure 2:**
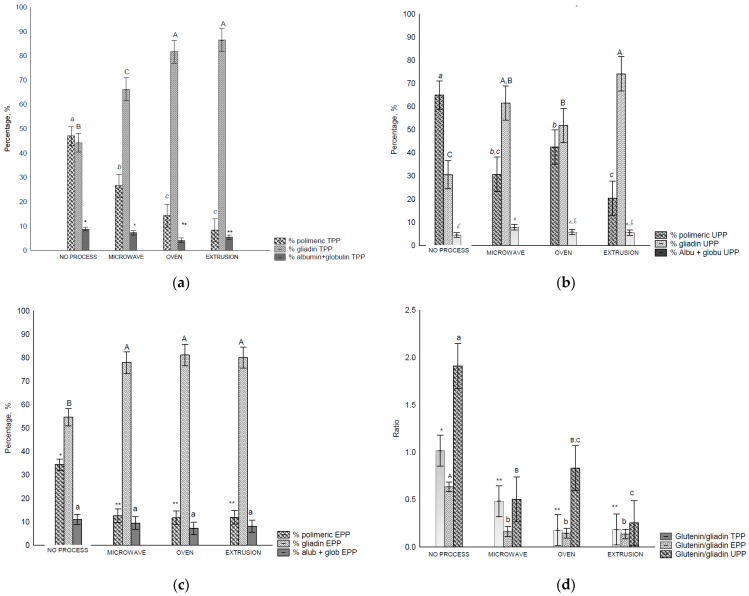
Size exclusion HPLC separation of total polymeric protein in control flour before and after treatments: (**a**)—total polymeric protein (TPP, %), (**b**)—unextractable polymeric protein (UPP), (**c**)—extractable polymeric protein (EPP), and glutenin/gliadin ratios extract from all fractions (TTP, UPP and EPP), (**d**)—Glutenin/gliadin ratios for TPP, UPP and EPP. Different letters (uppercase/lowercase) or symbols indicate different means according to Tukey’s test (*p* < 0.05). Vertical bars indicate deviation. No process = control flours.

The amounts of TPP (total polymeric protein) (Figure 2a), UPP (unextractable polymeric protein) (Figure 2b), EPP (extractable polymeric protein) (Figure 2c), and Glu/Glia ratios (Figure 2d) were affected by the treatments. When the TPP of the control flour was extracted, the proportions of polymeric and monomeric gliadins were not significatively different; however, after treatment, they were altered (Figure 2a–c). The number of polymeric proteins decreased, while that of the monomeric proteins increased (*p* < 0.05), most notably in the oven and extrusion treatments. Consequently, the Glu/Glia ratio (Figure 2d) decreased after all treatments.

In the UPP fraction of the control flour, polymeric proteins were found in higher amounts than the monomeric proteins owing to their higher complexity and, consequently, lower solubility. A decrease in the polymeric proteins was observed after all treatments (Figure 2b), and consequently the Glu/Glia ratios (Figure 2d) as the amount of extracted monomeric proteins increased. UPP comprises polymeric glutenin protein (>158 kDa) with the lowest solubility, and therefore, the highest molecular weight [28,42]. It is also related to the size and/or complexity of the gluten polymer [28] and the total number of HMW subunits [7]. In this study, we observed that the treatments affected the solubility of this fraction.

More monomeric proteins are present in the EPP (extractable polymeric protein) fraction. Nevertheless, after treatment, the proportion of monomeric proteins increased under all conditions (Figure 2c). In addition, decreased glutenin levels were observed. In all fractions, the polymeric proteins and Glu/Glia ratio decreased compared with the control flour which suggests poor rheological properties [12], affecting dough development and stability [43].

The data showed that the treatments modified the size and/or complexity of gluten proteins, resulting in more insoluble protein.

It was expected that post all treatments, polymeric glutenins would be the predominant protein group in the UPP fraction. However, this was not the case because of the external heating, shear, pressure, and radiation that were applied. Notably, monomeric gliadins were more abundant in EPP and TPP compared with the control flour.

During extrusion, the high temperature applied to proteins exposes the hydrophobic groups on the protein surface, resulting in interactions with other food components, causing a decrease in protein solubility [44]. The main structural changes during polymerization occur owing to isopeptide aggregation, Maillard reactions, and free-radical-initiated cross-linking, creating an anisotropic product that resembles meat-like textures, which may be desirable in some products. This is a direct result of aggregation and degradation, which promote modifications in the secondary, tertiary, and quaternary structures of the protein [44].

### 3.4. Glutenin and Gliadin Protein Characterization Using RP-HPLC

#### 3.4.1. Glutenins

RP-HPLC glutenins can be classified into HMW-GS and LWM-GS. Based on these, they can be characterized using an HMW-GS/LMW-GS ratio, whereby changes in the fractions (increase or decrease in extractability after treatment) can be measured.

**Figure 3:**
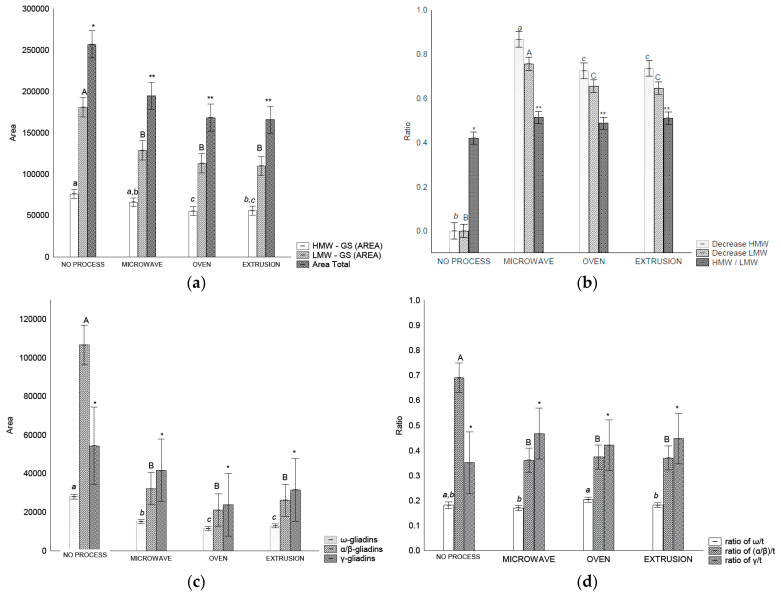
RP-HPLC separation of glutenins and gliadin in control flour before and after treatments: (**a**) area of total glutenins, HMW-GS and LMW-GS, (**b**) ratios between HMW-GS control flour/HMW-GS treatment, LMW-GS flour/LMW-GS treatment, and HMW/LMW-GS, (**c**) area of each gliadin fraction, (**d**) relation of gliadins fraction and total gliadin. Legend: Different letters (uppercase/lowercase) or symbols indicate different means according to Tukey’s test (*p* < 0.05). Vertical bars indicate the standard deviation. No process = control flour.

The extractability of HMW-GS decreased after oven and extrusion treatments compared to that of the control flour and microwave treatment (Figure 3a). For LMW-GSs and total glutenin, a decreased extractability (*p* < 0.05) was observed after all the treatments (Figure 3b).

HMW-GSs are directly related to the technological applications of wheat, as they are major determinants of dough elasticity [45]. In addition, HMW-GSs are required for glutenin formation, and affect the internal structure of glutenin [7].

The HMW-GS/LMW-GS ratio changed after all treatments, indicating an increase in glutenin size and complexity. These results are in accordance with those of [7] who observed that an alteration in the HMW/LMW-GS ratio is indicative of alterations in glutenin particle size.

During heat and mechanical treatments, the gluten protein unfolds, and protein cross-linking increases because of the exposure of hydrophobic regions and free SH groups that interact with each other. This leads to irreversible protein aggregation and the formation of a three-dimensional network of high molecular weight and viscous wheat gluten aggregates [39,46]. In addition, an increase in pressure and temperature leads to a significant reduction in the solubility and thiol content of gluten, gliadin, and glutenin, strengthening them [44].

#### 3.4.2. Gliadins

Gliadin distribution measured using RP-HPLC in wheat flour before and after treatment varied depending on the treatment (Figure 3c). It was possible to separate the gliadins into ω-, α/β- and γ-gliadin. The amount of ω-gliadin and α/β-gliadins extracted decreased (*p* < 0.05) after all treatments in comparison with the control flour. However, the γ-gliadins were not affected (*p* > 0.05) by any treatment (Figure 3c). In relation to the gliadin ratios, treatments affected the α/β-/t ratios that decreased after treatment (*p* < 0.05) (Figure 3d).

In baked products, gliadins are correlated with dough strength, mixing tolerance, and loaf volume [47]. During mixing and baking, the gliadins’ interchain S-S bonds start to form at 70 °C [22]. Heat and mechanical work cause α-, β-, and γ–gliadins (S-rich) to be incorporated into the gluten polymer with intermolecular SS bonds. Notably, ω-gliadins (S-poor) interact with hydrogen or other noncovalent bonds [4], altering the solubility and extractability of the flour. This results in changes in gliadin and glutenin distributions.

The extractability of gliadins in the control flour was higher than that of the treated samples (Figure 3c). Similar results have been reported with flour and bread, with S-S interaction attributed to this higher extractability; α- and γ-gliadins are more affected than are ω-gliadins [22,48]. Microwave heating can affect gliadin structure, leading to a decrease in gliadin extractability and an increase in the immunoreactivity by promoting conformational and chemical changes in the gliadin structure according to the level of energy applied, which results in an unsolved high molecular weight product in the chromatograms [19]. The same authors [19] reported that after microwave treatment, the content of all gliadin fractions decreased. Notably, we observed these decreases in ω-gliadin and α/β- gliadins. A similar gliadin distribution was noted in wheat from Argentina [47].

Microwave energy decreases the solubility and emulsifying capacity of gluten proteins, and the quality of baking value during baking tests [38]. Damage to gluten proteins is caused by an increase in temperature and irradiation, resulting in changes in protein structure and solubility [38].

A strong decrease in the amount of all gliadin fractions in microwave-irradiated wheat flour with an increase in applied energy was reported [19]; however, further studies showed a decrease in gliadin extractability [49].

High-pressure processing, such as extrusion, affects gluten by unfolding the proteins, partially denaturing and dissociating polymeric structures into subunits due to weakened electrostatic and hydrophobic bonds, causing the ionization of acid groups on amino acid side chains, and ultimately causing the aggregation and formation of gel networks or precipitates, resulting in poor rheological properties [50]. However, in some products, structural changes are desirable; the extrusion products can be flakes or meat-like products depending on the conditions, and this texture is due to the processes of denaturation, dissociation, and fragmentation, allowing the unraveled protein to align in the direction of shear [44].

### 3.5. Fourier Transform Infrared (FTIR) Spectroscopy

FTIR was used to assess the changes in the protein secondary structure caused by different treatments. The absorption bands represent the functional groups. Specifically, the amide I band corresponds to the C=O of the peptide bonds, which are determined by the secondary structure (α-helix, β-sheet, etc.). Treatments altered the secondary structure of wheat proteins (Table 2). In the general spectra, there was a flour-specific peak (not observed with the pure protein) at 1770–1732 cm^−1^, possibly determined by the extent of starch–protein interactions.

We observed a decrease (*p* < 0.05) in α-helix and random structures with treatment as compared to the control (Table 2). Mahroug et al. (2019) [51] also reported a decrease in α-helix structures after microwave treatment, suggesting that the heat induced to a sulfhydryl–disulfide interchange reaction resulted in a different arrangement of the disulfide bonds. In addition, the data showed an increase in β-sheets, β-turns, and other structures (Table 2). The high pressure and temperature of the extrusion process resulted in the largest decrease in the α-helix structure. This corroborates with the literature that showed that protein aggregation is primarily accompanied by the disappearance of α-helices and an increase in antiparallel β-sheets [44]. This is related to the higher stability of the β-sheet structures than α-helices in high-pressure-denatured proteins [50,52]. Compared to glutenin, gliadin is less affected by pressure and heat treatments because of its low thiol content. Meanwhile, compared with ω-gliadins (ω5- and ω1,2-gliadins), α- and γ-gliadins are more sensitive to high pressures, and intrachain disulfide bonds of α- and γ-gliadins are converted to interchain bonds [53].

Previous studies revealed that high pressure and medium temperature treatment on gluten protein led to a decrease in the β-sheets (%), antiparallel β-sheets (%), and α-helix (%) structures, and an increase in random structures [54], while microwave treatment could change β-turn structures to random structures [51].

Gluten contains large amounts of glycine, proline, glutamine, and leucine, which are the main contributors to hydrophobic interactions [55]. Most interactions are covalent (disulfide bonds), noncovalent (hydrogen, ionic, and hydrophobic bonds), and other bonds that are susceptible to modification. Among the physical modifications, heating–freezing and extrusion exhibit significant modifications to the gluten structure via the formation and dissociation of covalent bonds and noncovalent interactions [38].

Heating can induce sulfhydryl–disulfide interchange reactions, which involve the exchange between free thiol (-SH) groups and disulfide bonds (-S-S-) within the protein structure. This exchange can lead to a reorganization of disulfide bonds, potentially altering the protein’s secondary structure [51].

The increase and/or decrease in the relative abundance of certain secondary structures such as β-sheets or α-helices after heat treatment suggests that the protein’s conformation has changed. This rearrangement is evidenced by an observed increase in the relative abundance of specific secondary structures, β-sheets, indicating a shift in the protein’s conformation. In the HMW-GS, the main secondary structures were proposed to be β-turn organized in a regular β-spiral structure, and these are closely associated with the elastic behavior of gluten [56,57]. In the context of gluten, these structural modifications could impact its functional properties, such as elasticity, viscosity, and dough-forming ability, which are critical in various food applications.

### 3.6. Protein Digestibility

In addition to the important and fundamental technological qualities of wheat gluten proteins, there are concerns regarding their nutritional aspects and how these treatments affect digestibility. In this study, only extrusion showed a small yet significant decrease in digestibility (*p* < 0.05) compared to the control flour (Table 1). The nutritional value of a protein depends on its quantity, digestibility, and the availability of essential amino acids. The extrusion process was expected to improve the digestibility of proteins by inactivating protease inhibitors and other anti-physiological substances [58].

### 3.7. Immunoreactivity Using ELISA R5 and G12 Antibody Tests

The results showed a difference between the epitope availability for R5 and G12 antibodies. Using the R5 antibody, there was no statistically significant difference between the control and treatments (Table 1). When the G12 antibody was used, a reduction in recognition was observed in both oven and microwave treatments (Table 1). Oven and microwave treatments resulted in a 22% and 46% reduction, respectively.

The microwave radiation effect on the immunoreactivity of gluten was demonstrated by increasing the energy input but it showed the same effect as untreated flour after the highest power (500 W) and time (5 min) [19]. The same author also noted that microwave and heat treatment are closely related in regard to the changes in the immunoreactivity of gluten to celiac antibodies. This can be explained by the change in protein configurations that result in lower solubility.

Our results presented a decrease in α/β- and ω-gliadin extractability after microwave treatment (Figure 3c). A positive correlation between ω-gliadins, γ-gliadins, and total gliadin contents and immune reactivity to the R5 ELISA test, and no correlation to α/β-gliadin, has already been reported in the literature [59].

Recent attention has focused on the Italian patented product GlutenFriendly^TM^ technology [21] that uses microwave radiation to generate safe celiac flour that retains functionality. A recent report [49] demonstrated that, although the microwave process abolishes the recognition of epitopes by the R5 antibody, this is due to the protein insolubility and does not affect the immunological response to enzymatically digested microwave-treated gluten. Our results are consistent with those previously reported.

The commercial antibodies used herein bind to gluten-responsive DQ2/DQ8 T cell epitopes in celiacs [60,61]. The R5 monoclonal antibody recognizes the QQPFP repetitive pentapeptide epitope [61] and is recommended by the Codex Alimentarius. The G12 monoclonal antibody recognizes the QPQLPY repetitive hexapeptide epitopes primarily present in alpha gliadin [60].

In autoimmune diseases triggered by gluten, such as in celiac disease, gluten ataxia, and dermatitis herpetiformis, different epitopes are responsible for different presentations [62]. There are more than 50 T cell stimulatory peptides in gluten proteins, with varying degree of similarities, such as hydrophobic residues at specific positions and the higher toxicity of ω-gliadin [16], as well as in HMW-GS and LMW-GS [63].

It was observed that in vitro immunoreactivity against R5 and G12 antibodies showed a change based on the changes in glutenin and gliadin profiles (HMW-GS, LMW-GS, γ-, α/β-, and ω-gliadin) (Table 1). The observed changes in solubility and protein profiles suggest that the treatments modified the structure/complexity of gluten proteins, probably by masking epitopes and/or domains of glutenins and gliadins, leading to the decreased binding of G12 antibodies. Antibodies bind to specific protein sequences that are affected by new covalent and noncovalent interactions (i.e., hydrophobicity) induced by flour treatments.

The manner in which gluten proteins are presented to individuals may be related to disease development. Further research could clarify whether, together with genetic conditions, the way in which gluten protein reaches the gut defines how and if an autoimmune disease will manifest.

Gliadins are primarily detected as toxic to celiacs, and there has been an increase in the development of methods for detecting traces of gliadin in heat-treated and non-heat-treated foods. The current market includes several ELISA kits for antibodies directed against the epitopes of gliadin, which are toxic to celiac people. It should also be noted that, except for HMW- GS, disulfide, tryptophan, and tyrosine bonds may also exist in gliadins and/or LMW-GSs, which may affect dough properties [12].

## 4. Conclusions

The effects of different processing conditions on the gluten network extractability, digestibility secondary structure, and antibody recognition were investigated in this study. The results indicate a decrease in the solubility of the polymeric and monomeric proteins. In addition, the treatments affected the glutenin and gliadin profiles; glutenins become less extractable with an increase in gliadin extractability. These changes are the result of the rearrangement of proteins during the treatments, resulting in a more complex, less soluble structure. FTIR analysis revealed that changes in protein secondary structure are involved in the observed changes in extractability, with significant increases in the intermolecular β-sheet and β-turns and a decrease in α-helix being observed for all treated samples compared to the control flour. Protein digestibility remained unchanged, except for the extruded sample, which showed a small but significant decrease in digestibility, most likely due to the high temperature and pressure conditions. The potential celiac disease immune stimulatory epitopes were measured and found to be decreased in oven and microwave treatment by the G12 ELISA; however, no change was observed using the R5 antibody. These findings illustrate the structural and physicochemical changes in wheat proteins during heating and microwaving. Understanding the effects of various flour treatments might be beneficial to the production of specific modified flours to develop improved wheat-based foods.

## Figures and Tables

**Table 1:** Total protein, soluble, and insoluble polymeric protein (%), protein digestibility (%), and in vitro immunoreactivity of wheat flour before and after treatment.

Treatment	TP * (%)	IPP ** (%)	SPP *** (%)	Digestibility (%)	R5 (g/100 g)	G12 (g/100 g)
Control Flour	12.1 ± 0.3 ^a^	5.6 ± 0.2 ^c^	6.5 ± 0.2 ^a^	95.60 ± 0.54 ^a^	9.64 ± 0.38 ^a^	10.78 ± 0.44 ^a^
Microwave	12.4 ± 0.2 ^a^	9.1 ± 0.6 ^b^	3.3 ± 0.5 ^b^	96.52 ± 0.36 ^a^	11.13 ± 0.07 ^a^	5.75 ± 0.11 ^c^
Oven	12.2 ± 0.2 ^a^	10.7 ± 0.2 ^a^	1.5 ± 0.2 ^c^	95.70 ± 0.97 ^a^	10.89 ± 1.35 ^a^	8.42 ± 0.83 ^b^
Extrusion	12.0 ± 0.2 ^a^	9.8 ± 0.3 ^ab^	2.3 ± 0.1 ^d^	92.72 ± 6.04 ^b^	12.11 ± 1.02 ^a^	10.58 ± 0.02 ^a^

* TP = total protein; ** IPP = insoluble polymeric proteins; *** SPP = soluble polymeric proteins. Different letters within the same column indicate a significant difference (*p* < 0.05). Values are presented as the mean ± standard deviation.

**Table 2:** FTIR peak positions and distribution of protein structure in wheat flour before and after treatments (percentage, %).

Protein Structure		Peak Position	Control Flour	Microwave	Oven	Extrusion
β-turn	β-turn 1	1688 ± 2	20.4 ± 0.6 ^b^	27.3 ± 1.5 ^a^	26.1 ±3.4 ^a^	28.1 ± 1.6 ^a^
	β-turn 2	1674 ± 2				
α-helix		1659 ± 1	32.1 ± 0.9 ^a^	17.1 ± 1.4 ^bc^	21.5 ±1.1 ^b^	16.6 ± 1.2 ^c^
Random		1649 ± 1	16.8 ± 0.8 ^a^	12.5 ± 1.2 ^bc^	14.6 ±1.1 ^b^	11.2 ± 1.3 ^c^
β-sheet	β-sheet 1	1641 ± 1	26.2 ± 1.2 ^b^	32.7 ± 2.1 ^a^	29.3 ±3.2 ^a^	32.8 ± 1.7 ^a^
	β-sheet 2	1629 ± 1				
Other		1611 ± 2	4.5 ± 0.5 ^c^	10.4 ± 1.1 ^ab^	8.5 ±1.6 ^b^	11.3 ± 0.8 ^a^

Different letters within the same row indicate a significantly different (*p* < 0.05). Values presented as mean ± standard deviation.

## Data Availability

The original contributions presented in the study are included in the article, further inquiries can be directed to the corresponding author. Public data will be added to the USDA-NAL Ag Data Commons data repository.
